# Relative Validation of an Artificial Intelligence–Enhanced, Image-Assisted Mobile App for Dietary Assessment in Adults: Randomized Crossover Study

**DOI:** 10.2196/40449

**Published:** 2022-11-21

**Authors:** Audrey Moyen, Aviva Ilysse Rappaport, Chloé Fleurent-Grégoire, Anne-Julie Tessier, Anne-Sophie Brazeau, Stéphanie Chevalier

**Affiliations:** 1 School of Human Nutrition McGill University Sainte-Anne-de-Bellevue, QC Canada; 2 Research Institute of the McGill University Health Centre Montreal, QC Canada; 3 Department of Medicine McGill University Montreal, QC Canada

**Keywords:** dietary intake, dietary assessment, food diary, food records, automated self-administered 24-hour recall, ASA24, Keenoa

## Abstract

**Background:**

Thorough dietary assessment is essential to obtain accurate food and nutrient intake data yet challenging because of the limitations of current methods. Image-based methods may decrease energy underreporting and increase the validity of self-reported dietary intake. Keenoa is an image-assisted food diary that integrates artificial intelligence food recognition. We hypothesized that Keenoa is as valid for dietary assessment as the automated self-administered 24-hour recall (ASA24)–Canada and better appreciated by users.

**Objective:**

We aimed to evaluate the relative validity of Keenoa against a 24-hour validated web-based food recall platform (ASA24) in both healthy individuals and those living with diabetes. Secondary objectives were to compare the proportion of under- and overreporters between tools and to assess the user’s appreciation of the tools.

**Methods:**

We used a randomized crossover design, and participants completed 4 days of Keenoa food tracking and 4 days of ASA24 food recalls. The System Usability Scale was used to assess perceived ease of use. Differences in reported intakes were analyzed using 2-tailed paired *t* tests or Wilcoxon signed-rank test and deattenuated correlations by Spearman coefficient. Agreement and bias were determined using the Bland-Altman test. Weighted Cohen κ was used for cross-classification analysis. Energy underreporting was defined as a ratio of reported energy intake to estimated resting energy expenditure <0.9.

**Results:**

A total of 136 participants were included (mean 46.1, SD 14.6 years; 49/136, 36% men; 31/136, 22.8% with diabetes). The average reported energy intakes (kcal/d) were 2171 (SD 553) in men with Keenoa and 2118 (SD 566) in men with ASA24 (*P*=.38) and, in women, 1804 (SD 404) with Keenoa and 1784 (SD 389) with ASA24 (*P*=.61). The overall mean difference (kcal/d) was −32 (95% CI −97 to 33), with limits of agreement of −789 to 725, indicating acceptable agreement between tools without bias. Mean reported macronutrient, calcium, potassium, and folate intakes did not significantly differ between tools. Reported fiber and iron intakes were higher, and sodium intake lower, with Keenoa than ASA24. Intakes in all macronutrients (*r*=0.48-0.73) and micronutrients analyzed (*r*=0.40-0.74) were correlated (all *P*<.05) between tools. Weighted Cohen κ scores ranged from 0.30 to 0.52 (all *P*<.001). The underreporting rate was 8.8% (12/136) with both tools. Mean System Usability Scale scores were higher for Keenoa than ASA24 (77/100, 77% vs 53/100, 53%; *P*<.001); 74.8% (101/135) of participants preferred Keenoa.

**Conclusions:**

The Keenoa app showed moderate to strong relative validity against ASA24 for energy, macronutrient, and most micronutrient intakes analyzed in healthy adults and those with diabetes. Keenoa is a new, alternative tool that may facilitate the work of dietitians and nutrition researchers. The perceived ease of use may improve food-tracking adherence over longer periods.

## Introduction

### Background

Associations among diet, health, and disease have been made, but findings are often criticized based on the unreliability of the collected dietary data. Indeed, current dietary assessment methods used in research are either burdensome to participants, resulting in a lower rate of compliance, or lack accuracy and precision [1]. They are also expensive because it is time-consuming for researchers to collect and analyze data. These methods include (1) written food diaries, which require participants to write every food and beverage consumed, usually during 3 to 7 consecutive days [2,3]; (2) 24-hour recalls where participants report all foods and beverages consumed for the previous day in the presence of a trained interviewer; and (3) food frequency questionnaires, which ask the frequency of consumption of specific food items or categories of food consumed during a defined time period [4]. Food recalls rely on memory for both the food items consumed and their quantity [4], which may impact the validity of collected data. One of the most common methods for dietary assessment is the food diary that is often incomplete and therefore requires researchers to make assumptions to evaluate dietary intake. Food diaries are more prone to reactivity bias; that is, when participants change their food intake owing to the awareness of their intake being analyzed [4]. Lack of participant motivation may also be a source of error, limiting the number of valid tracking days [4,5]. Furthermore, research personnel must be trained on standardized methods to administer, review, and analyze food diaries. This is of particular importance in the context of multisite studies. In addition, these methods require participants to estimate their food portion sizes accurately and are demanding on research personnel who must enter collected dietary data into computer software for data analysis. Finally, underestimation of total calorie intake is frequent, in particular among individuals living with overweight and obesity [3,6,7].

New tools have been developed using web and mobile technologies [8-11]. It is now possible to collect food diaries and recalls on the web, to facilitate the recording process and data analysis [9]. Only a few image-assisted food record methods exist, and few studies have investigated their use. Evidence points to potential improvement of accuracy with such methods compared with traditional self-reported dietary assessment approaches by providing through pictures of meals, food items, and additional details that could be otherwise omitted [12-14]. The use of pictures may decrease underreporting and increase the accuracy of portion size estimation. Keenoa is a newly designed, image-assisted food-tracking mobile app that integrates artificial intelligence for food recognition. It was developed to introduce user-friendly technologies facilitating the work of researchers and dietitians. Given the lower burden on participants, it has the potential to overcome some of the limitations of traditional methods.

### Objectives

This study was conducted to evaluate the relative validity of Keenoa against a 24-hour validated web-based food recall platform automated self-administered 24-hour recall (ASA24) in both healthy individuals and those living with type 1 and type 2 diabetes. People with diabetes, namely type 1, are generally more aware of their food intake to match with insulin injections, hence we aimed to validate the tool in both a healthy and diabetic population. Secondary objectives were to compare the proportion of under and overreporters between tools and to assess the user’s appreciation of the tools. We hypothesized that dietary assessment using Keenoa is comparable with that of ASA24-Canada and better appreciated by users.

## Methods

### Study Population

Participants were recruited between February and November 2021 through social media, email, and word of mouth. Participants with diabetes were recruited through the Behaviors, Therapies, Technologies, and Hypoglycemic Risk in Type 1 Diabetes (BETTER) registry [15] and through emails from a diabetes association (ie, Diabète Québec). Inclusion criteria were adults aged 18 to 70 years; owning a smartphone, computer, or tablet; having access to the internet; being able to read English or French; and having a self-reported BMI between 18 and 35 kg/m^2^ to minimize under- and overreporting [16]. Exclusion criteria were living outside of Canada, history of frequent dieting, following a weight loss diet, having a disordered eating pattern, having any active and uncontrolled acute or chronic disease, having gained or lost a significant amount of weight (>5 kg) within the past 3 months, and being pregnant or breastfeeding. Participants with diabetes were excluded if their diagnosis dated ≤1 year, if their diabetes medication was changed within the past 3 months or if they had celiac disease. Electronic informed consent was obtained from all participants.

### Ethics Approval

The study was approved by the McGill Research Ethics Board (REB 20-09-035) and registered on the Dietary Assessment Calibration or Validation Register from the National Cancer Institute.

### Study Design

We used a randomized crossover design for a 2-week duration, and participants completed a 4-consecutive-day tracking period (3 weekdays and 1 weekend day) with both dietary assessment methods, ASA24 and Keenoa, in a random order, on the same days of the week. The study was performed entirely on the web. Participants were also asked to answer a web-based questionnaire about their sex, gender, height, weight, years of education, occupation, chronic diseases, weight history, body image satisfaction, medications, and vitamin and mineral supplements (Multimedia Appendix 1). The questionnaire also included questions on physical activity levels following the International Physical Activity Questionnaire–Short Form [17]. Participants were given written instructions (1 page each) on the use of each tool, and assistance was available as needed by email or phone to ensure accurate data entry. Upon completion of the questionnaire, they were assigned a set of 4 consecutive days to track their food intake. Those 4 days were determined to begin on the Wednesday or Sunday following questionnaire completion to allow for the 4 consecutive days to include 3 weekdays. If patients omitted to enter their daily food intake, reminders were sent at the end of the day, before dinner time. With ASA24, participants could still log in until midnight to fill their recall. If the recall had not been completed by the next day, participants were allotted another day of tracking (weekday or weekend day, depending on the missing day). With Keenoa, missing days were also replaced with additional tracking days. Each participant was allotted a maximum of 2 reminders per tool, after which they were excluded from the study. An encouragement message was sent after the completion of the first day with each tool to maximize adherence. Finally, participants were asked to complete the System Usability Scale (SUS) [18], a validated usability questionnaire that consists of 10 questions with 5 options each, from strongly disagree to strongly agree. The SUS asked participants about both tools in the same order in which they tracked their intake (ie, if participants used Keenoa first and ASA24 second, the tool asked participants to answer questions about Keenoa followed by the same set of questions on ASA24). After completing the tracking period with both dietary assessment tools, if food items present in the pictures of participants were omitted or entered incorrectly, entries from the Keenoa app were adjusted by a dietitian. For instance, when condiments such as ketchup or mayonnaise were present in a picture but not in the participants’ entries, the item was added to the food diary.

### Automated Self-administered 24-Hour Recall

The ASA24 is a web-based dietary recall or food diary tool developed by the National Cancer Institute of the National Health Institute designed for epidemiological and clinical research purposes and is free to use. The ASA24 recall tool has been extensively validated notably against true intake [19] and recovery biomarkers [20], hence we used the recall tool as a comparator. The recall tool was designed to mimic a 24-hour recall without requiring an interviewer. As such, it uses the multiple-pass method which includes multiple reminders on frequently forgotten foods or ingredients and asks for details regarding portion sizes using pictures as models. Once completed, researchers access the data analysis, which is based on the Canadian Nutrient File (CNF; version 2015) for the ASA24-Canada 2018. The ASA24 tool allows to enter supplements, with an option to include them in the analytic files. In this study, supplements were not included in the nutrient comparisons.

### Keenoa

Keenoa (version 1.0.3) is a newly designed, intelligent food-tracking mobile app (participant’s end) linked to a web platform (researcher’s end). With the mobile app, participants take pictures of their meals and snacks, which are recognized or partly recognized by an artificial intelligence–based algorithm. Prompted by a few questions, users specify the foods and beverages consumed and estimate portion sizes using dynamic pictograms. From the web app, the researcher has access to the meal pictures and their associated detailed nutritional analysis, in real time. Dietary data are obtained from the CNF database [21], the US Department of Agriculture database [22], and frequently imported food items from the South Korean Food Composition Database [23], the Hong Kong Nutrient Information Inquiry System [24], the Indian Food Composition Tables [25], and the Australian Food Composition Database [26]. Bar codes from grocery store items can be scanned from the app. Relevant information such as time of consumption and time between meals is also available. The Keenoa app has been validated in the past [27,28]. However, those were performed using a prototype of the app. The validity and reliability of the updated Keenoa food diary remain to be established for both research and evidence-based dietetic practice.

### Sample Size

Considering that correlations between reference and test methods are expected to range between 0.5 and 0.7 in the context of validity, based on a 0.4 null-hypothesis correlation coefficient, 0.6 effect size (alternative hypothesis), Cronbach α=.05, and 80% power, the sample size required was 111 participants. This sample size for dietary intake validation studies is supported by Serra-Majem et al [29] and Willett [4]. Thus, we aimed to recruit 120 healthy participants within 3 age strata of 18 to 35, 36 to 54, and 55 to 70 years with an equal gender ratio of 20 (50%) men and 20 (50%) women per stratum. In addition, we aimed to recruit 40 patients (20 men and 20 women, 18-70 years) with diagnosed type 1 diabetes and 40 patients (20 men and 20 women, 18-70 years) with diagnosed type 2 diabetes for this study.

### Statistical Analysis

Energy and macronutrient intakes were averaged to reflect habitual intake. Mean results from Keenoa were compared with those from ASA24 using paired sample 2-tailed *t* tests and Wilcoxon rank tests, by gender, with and without adjustment for caloric intake as per the residuals method [30]. The normality of data distributions was evaluated using the Shapiro-Wilk test. Correlations were evaluated using Spearman correlation coefficients and were deattenuated, as suggested [31]. Deattenuation involves adjusting for reliability, by dividing a correlation by the product of the 2 tools’ daily intraindividual variability [32] from intraclass correlations. Weighted Cohen κ values were computed to assess interrater agreement between tools and were interpreted as per Landis and Koch [33]. The Bland-Altman method was used to assess the mean difference and agreement between the 2 tools. We established our acceptable limits of agreement (LOAs) as the mean intraindividual SD between reported daily intakes multiplied by 1.96, given that LOAs in similar studies are often large and no acceptable LOAs have been established in the literature for nutrition validation studies. Pearson correlations were used to assess associations between differences and average intake with Bland-Altman plots, as suggested by Lombard et al [31]. Energy underreporting was defined as a ratio between mean 4-day energy intake and estimated resting energy expenditure ratio below 0.9 [34] and overreporting as a ratio >2.4, as previously established [35]. Resting energy expenditure was estimated using the Mifflin St-Jeor equation [36]. The significance of differences in under- and overreporting was measured using χ^2^ tests. All analyses were performed using SPSS (version 28; IBM Corp). *P* values <.05 were considered to be statistically significant.

## Results

### Participant Characteristics

**Figure 1:**
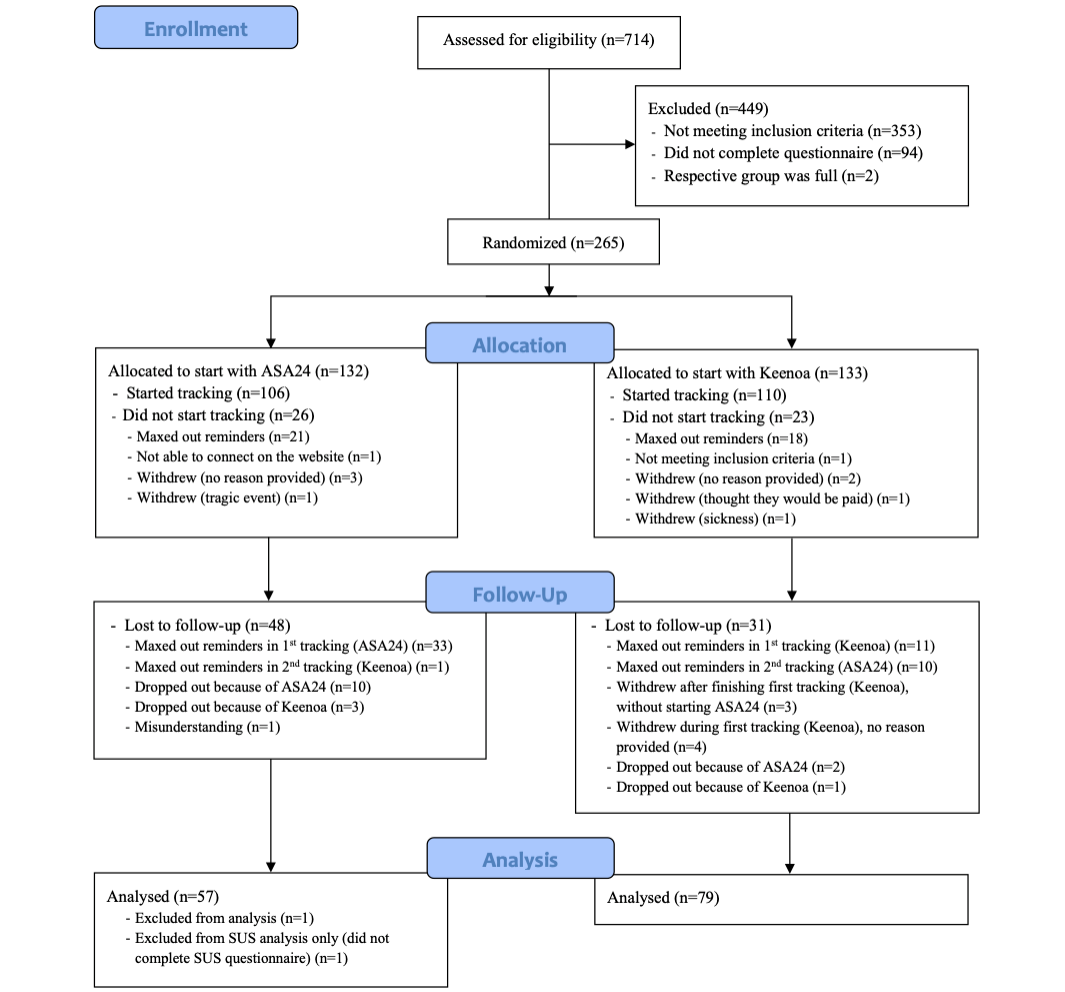
Participant flow. ASA24: automated self-administered 24-hour recall; SUS: System Usability Scale.

A total of 361 respondents were eligible to participate in the study. Of the 361 participants, 92 (25.5%) participants did not fill the first questionnaire, 2 (0.5%) were screened when their respective stratum was full and were therefore excluded, 128 (35.4%) dropped out before completing both dietary tracking periods, and 1 (0.3%) participant was excluded owing to outstanding differences between both tracking periods (2.25-fold difference in energy intake). Completion rates and dropouts varied by tool with more dropouts and exclusions during ASA24 tracking (55/128, 43%) as opposed to Keenoa tracking (16/128, 12.5%; Figure 1). In total, 136 participants were included in the analyses; 11% (15/136) of participants were trained in nutrition or dietetics. Baseline participant characteristics are summarized in Table 1, by gender. The mean age of participants was 45.4 (SD 14.5) years in women and 47.2 (SD 15.2) years in men. The mean BMI was 24.0 (SD 3.9) kg/m^2^ in women and 26.8 (SD 4.4) kg/m^2^ in men. Most participants were of White ethnicity (113/136, 83.1%) and had a bachelor’s degree (101/136, 74.3%).

**Table 1:** Characteristics of study participants (N=136)^a^.

Variable		Women (n=87)	Men (n=49)
Age (years), mean (SD)		45.4 (14.5)	47.2 (15.2)
BMI (kg/m^2^), mean (SD)		24.0 (3.9)	26.8 (4.4)
**Diabetes,** **n (%)**			
	Yes	22 (25.3)	9 (18.4)
	No	65 (74.7)	40 (81.6)
**Chronic disease,** **n (%)**			
	Yes	30 (34.5)	12 (24.5)
	No	56 (64.4)	37 (75.5)
**Level of education, n (%)**			
	Some high school completed	1 (1.1)	1 (2)
	High school diploma	2 (2.3)	2 (4.1)
	Vocational school	1 (1.1)	4 (8.2)
	Completed college	18 (20.7)	3 (6.1)
	Bachelor’s degree	35 (40.2)	18 (36.7)
	Graduate degree (ie, MSc, MA, or PhD)	26 (29.9)	18 (36.7)
	Professional degree (ie, MD)	1 (1.1)	3 (6.1)
**Ethnicity, n (%)**			
	White	74 (85.1)	39 (79.6)
	Aboriginal (first nations)	1 (1.1)	1 (2)
	Asian	7 (8)	5 (10.2)
	Black	0 (0)	2 (4.1)
	Hispanic	3 (3.4)	2 (4.1)
	Multiethnic	1 (1.1)	0 (0)
**Body weight satisfaction** **, n (%)**			
	Yes	41 (47.1)	33 (67.3)
	No	38 (43.7)	15 (30.6)
	I do not know or I prefer not to answer	8 (9.1)	1 (2)
**Medication** **, n (%)**			
	Yes	52 (59.8)	25 (51)
	No	35 (40.2)	24 (49)
**Taking vitamin or mineral supplement** **, n (%)**			
	Yes	28 (32.2)	20 (40.8)
	No	59 (67.8)	29 (59.2)
**Special diets** **, n (%)**			
	None	67 (77)	41 (83.7)
	Vegetarian, vegan	8 (9.2)	3 (6.1)
	Intermittent fasting	1 (1.1)	0 (0)
	Gluten free	1 (1.1)	0 (0)
	Other (eg, Mediterranean or low carb)	10 (21.7)	5 (10.2)
**Cooking responsibility** **, n (%)**			
	Myself	78 (89.7)	32 (65.3)
	Another family or household member	7 (8)	17 (34.7)
**Grocery shopping responsibility** **, n (%)**			
	Myself	76 (87.4)	35 (71.4)
	Another family or household member	9 (10.3)	14 (28.6)
**Self-reported physical activity level** **, n (%)**			
	Sedentary	16 (18.4)	7 (14.3)
	Low active	34 (39.1)	20 (40.8)
	Active	35 (40.2)	21 (42.9)
	Very active	2 (2.3)	1 (2)
**Used diet tracking app before** **, n (%)**			
	Yes	32 (36.8)	17 (34.7)
	No	55 (63.2)	32 (65.3)

^a^All missing data not adding up to the total sample population are “I don’t know” or “I prefer not to answer.”

### Mean Differences and Agreement Between Tools

Mean reported intakes of energy and all selected nutrients are reported by the tool, presented in Table 2 for women and Table 3 for men. Mean reported energy intakes (kcal/d) with Keenoa and ASA24 were 2171 (SD 553) and 2118 (SD 566; *P*=.38) in men, and 1804 (SD 404) and 1784 (SD 389; *P*=.61) in women, respectively. There were no statistically significant differences between tools in macronutrient intake (all *P*>.05) except for fibers, which was higher with Keenoa in both women (*P*<.001) and men (*P*=.02). Differences in macronutrient distribution were also nonsignificant in both genders. From the micronutrients analyzed, reported potassium and folate intakes did not differ between tools. Reported fiber intakes were higher with Keenoa in both women (*P*=.002) and men (*P*=.05) in both genders. Mean reported calcium intake was not different between tools in women (*P*=.67) but men had significantly lower calcium with ASA24 with a mean difference of −90 mg/d (95% CI −175 to −5; *P*=.04). Reported sodium intake was higher with ASA24 in both genders (*P*<.001). Differences or the absence of differences remains when adjusting nutrients for energy intake (data not shown).

**Figure 2:**
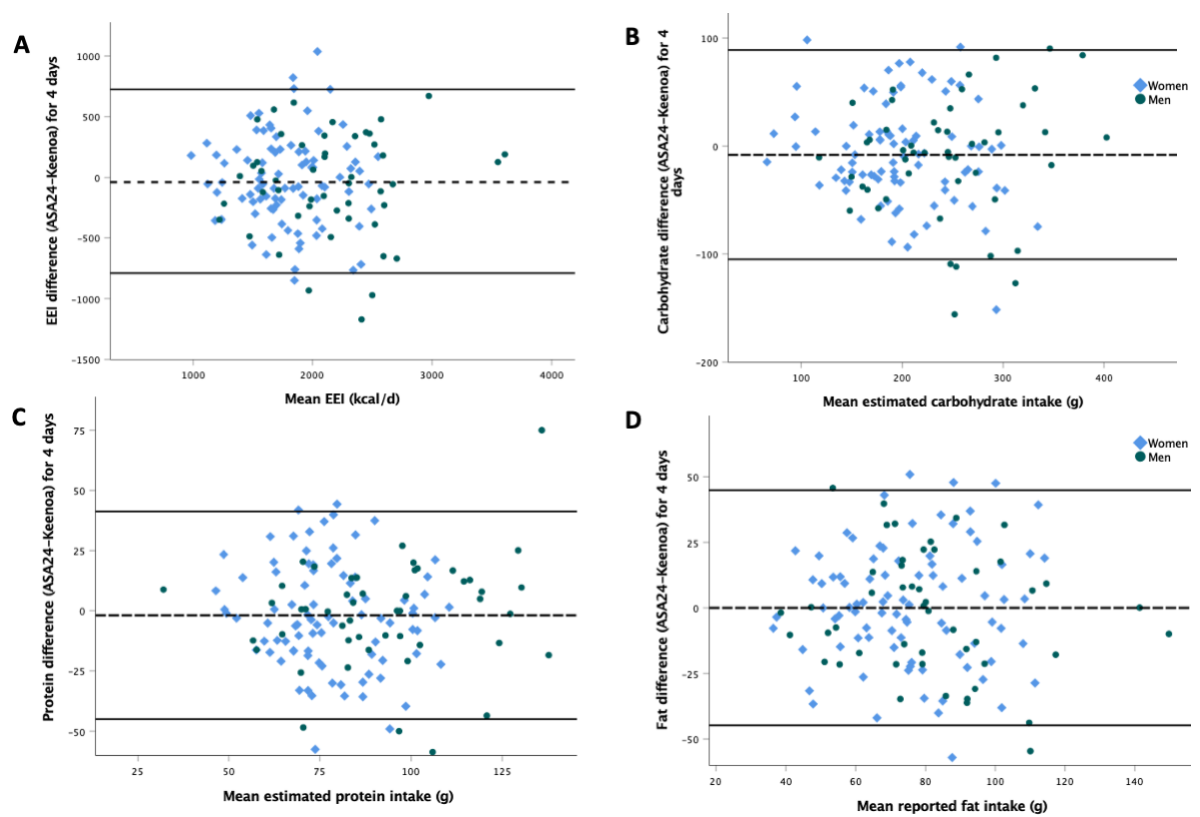
Bland-Altman plots. ASA24: automated self-administered 24-hour recall; EEI: estimated energy intake.

Results from Bland-Altman analyses are shown in Figure 2 and Table 4. Pooling participants of both genders resulted in mean differences of −32 kcal (LOAs: −789.2 to 725.2) for energy, −7.9 g (LOAs: −104.8 to 89.0) for carbohydrate, −1.9 g (LOAs: −45.0 to 41.3) for protein, and 0.0 g (LOAs: −44.8 to 44.9) for fat intakes, as shown in Bland-Altman plots (Figure 2); negative values indicated that reporting was higher with Keenoa compared with ASA24. Bland-Altman plots for energy and macronutrients by gender are presented in Figure 2. LOAs of all nutrients (Table 4) were within acceptable LOAs except for calcium, sodium, and folate, which had larger LOAs for the difference between tools as opposed to the intraindividual daily variability LOAs.

**Table 2:** Reported nutrient intakes and mean differences between automated self-administered 24-hour recall (ASA24) and Keenoa in women (n=87)^a^.

	ASA24, mean (SD)	Keenoa, mean (SD)	Mean difference (95% CI)	*P* value
Energy (kcal)	1784 (389)	1804 (404)	−20 (−99 to 58)	.61
Carbohydrates (g)	195.3 (55.8)	202.0 (63.5)	−6.6 (−16.5 to 3.2)	.19
Protein (g)	76.9 (17.6)	79.0 (19.7)	−2.1 (−6.8 to 2.5)	.37
Fat (g)	76.1 (22.6)	74.8 (21.6)	1.2 (−3.6 to 6.1)	.61
Fiber (g)	19.8 (7.6)	22.2 (9.0)	−2.4 (−3.7 to −1.1)	<.001
Calcium (mg)	860 (309)	875 (384)	−15 (−85 to 55)	.67
Iron (mg)	12.0 (3.2)	13.6 (5.1)	−1.6 (−2.5 to −0.6)	.002^b^
Sodium (mg)	2950 (739)	2442 (799)	508 (302 to 715)	<.001^b^
Potassium (mg)	2852 (755)	2929 (896)	−77 (−202 to 48)	.23
Folate (µg)	434 (129)	406 (135)	28 (−0.4 to 56)	.05^b^

^a^Paired 2-tailed *t* tests unless otherwise indicated.

^b^Wilcoxon signed-rank test.

**Table 3:** Reported nutrient intakes and mean differences between automated self-administered 24-hour recall (ASA24) and Keenoa in men (n=49)^a^.

	ASA24, mean (SD)	Keenoa, mean (SD)	Mean difference (95% CI)	*P* value
Energy (kcal)	2118 (566)	2171 (553)	−53 (−173 to 68)	.38
Carbohydrates (g)	239.1 (74.2)	249.3 (67.1)	−10.2 (26.0 to 5.6)	.20
Protein (g)	92.0 (27.3)	93.3 (24.0)	−1.4 (−7.8 to 5.1)	.67
Fat (g)	80.6 (24.1)	82.8 (27.5)	−2.1 (−8.8 to 4.5)	.52
Fiber (g)	21.7 (10.2)	24.3 (8.7)	−2.5 (−4.6 to −0.5)	.02
Calcium (mg)	874 (323)	964 (351)	−90 (−175 to −5)	.04
Iron (mg)	13.8 (4.2)	14.8 (4.7)	−1.0 (−2.0 to −0.0)	.05
Sodium (mg)	3534 (1146)	2880 (930)	654 (383 to 924)	<.001^b^
Potassium (mg)	3188 (1111)	3323 (1206)	−136 (−492 to 220)	.26^b^
Folate (µg)	475 (195)	511 (237)	−37 (−99 to 26)	.10

^a^Paired 2-tailed *t* tests unless otherwise indicated.

^b^Wilcoxon signed-rank test.

**Table 4:** Bland-Altman agreement analysis (N=136).

	Mean difference (SD)^a^	LOAs^b^	Acceptable LOAs^c^	Pearson *r*^d^	*P* value^d^
Energy (kcal)	−32.0 (386.3)	−789.2 to 725.2	−849.1 to 785.1	−0.022	.80
Carbohydrates (g)	−7.9 (49.4)	−104.8 to 89.0	−110.1 to 94.3	−0.048	.58
Protein (g)	−1.9 (22.0)	−45.0 to 41.3	−46.7 to 42.9	0.017	.84
Fat (g)	0.0 (22.9)	−44.8 to 44.9	−46.2 to 46.2	−0.047	.59
Fiber (g)	−2.4 (6.5)	−15.2 to 10.3	−16.6 to 11.8	−0.045	.60
Calcium (mg)	−42.1 (317.4)	−664.2 to 580.0	−588.4 to 504.2	−0.214	.01
Iron (mg)	−1.4 (4.1)	−9.5 to 6.7	−9.5 to 6.7	−0.341	<.001
Sodium (mg)	560.7 (959.0)	−1315.9 to 2440.4	−1126.6 to 2248.0	0.091	.29
Potassium (mg)	−97.9 (875.9)	−1814.8 to 1618.9	−1884.8 to 1689.0	−0.155	.07
Folate (µg)	4.7 (170.8)	−330.0 to 339.5	−321.2 to 330.6	−0.233	.006

^a^Automated self-administered 24-hour recall (ASA24)—Keenoa.

^b^LOA: limit of agreement.

^c^Established as the mean intraindividual SD between reported daily intakes multiplied by 1.96.

^d^Refers to the Pearson correlation between the mean and mean difference between the tools. Significant *P* values indicate systematic bias.

For energy and macronutrient intakes, deattenuated correlation coefficients ranged from 0.69 to 1.0 in men and from 0.42 to 0.84 in women (Multimedia Appendix 1). Micronutrients’ deattenuated correlation coefficients ranged from 0.61 to 1.0 in men and 0.31 to 1.0 in women. Sodium was the nutrient with the lowest correlation coefficient value in women.

### Cross-Classification Analysis

Results from the cross-classification analysis are shown in Table 5. The weighted κ score associated with energy reporting with both tools was 0.45 (95% CI 0.34-0.56). Weighted κ scores ranged from 0.29 to 0.52 for macronutrients and from 0.31 to 0.51 for the micronutrients analyzed (all *P*<.001).

**Table 5:** Cross-classification agreement by quartiles.

	Cohen κ^a^ (95% CI)	*P* value^b^
Energy (kcal)	0.45 (0.34-0.56)	<.001
Carbohydrates (g)	0.52 (0.42-0.62)	<.001
Protein (g)	0.29 (0.17-0.42)	<.001
Fat (g)	0.32 (0.20-0.43)	<.001
Fiber (g)	0.51 (0.41-0.61)	<.001
Calcium (mg)	0.38 (0.27-0.49)	<.001
Iron (mg)	0.38 (0.26-0.49)	<.001
Sodium (mg)	0.31 (0.19-0.43)	<.001
Potassium (mg)	0.49 (0.39-0.60)	<.001
Folate (µg)	0.37 (0.25-0.48)	<.001

^a^Weighted Cohen κ.

^b^Significant *P* values indicate agreement.

### Usability

Usability measured by the SUS indicated a mean score of 52.9 (SD 21.3) points out of 100 for ASA24 and 77.0 (SD 16.2) points for Keenoa (*P*<.001). Using the star rating (out of 5) after the completion of both tracking periods, of the 136 participants, 101 (74.8%) participants rated Keenoa higher compared with ASA24, whereas 11 (8.1%) gave a higher rating to ASA24. Among the 136 participants included in the analysis, 33 (24.3%) needed 2 reminders before they completed ASA24, while 11 (8.1%) needed 2 reminders with Keenoa. More reminders were needed during the first tracking with either tool. Finally, the frequency of energy underreporting was the same for each tool (12/136, 8.8%), and no overreporting was observed.

## Discussion

### Principal Findings

This study assessed the relative validity of the Keenoa mobile app against the validated ASA24 web-based recall method. No significant differences in estimated energy intake were found between tools that had similar rates of energy underreporting. Mean carbohydrate, protein, fat, potassium, and folate intakes were also similar between the 2 tools, whereas fiber and iron reporting was higher and sodium was lower with Keenoa than ASA24, in both genders.

### Interpretation of Findings

Bland-Altman analyses and paired 2-tailed *t* tests showed no systematic bias and agreement for all macronutrients and energy, with LOA falling within acceptable values, and without significant trends in the differences per intake. Weighted Cohen κ scores, which represent the ability of the tools to classify participants similarly based on their nutrient intake, indicated fair (fat and protein) to moderate (energy and carbohydrates) agreement at the individual level.

In addition to the main macronutrients and energy, fiber, calcium, iron, sodium, potassium, and folate were selected for analysis because they typically show little variability in day-to-day consumption [37,38] while generally of interest to clinicians and researchers. No systematic bias was detected for potassium and folate and calcium at a group level. Fiber and iron were consistently higher with Keenoa compared with ASA24, although the magnitude of the difference was relatively small: 2.4 g and 1.4 mg, respectively, both 11% of the mean estimated intake. On the other hand, reported sodium intake was consistently higher with ASA24, with a larger difference of 561 mg (19% of mean estimated intake). Fiber, iron, and potassium had LOAs lower than intraindividual daily variation LOAs suggesting an acceptable level of uncertainty between the tools. LOA for calcium, sodium, and folate were wider than the acceptable LOAs, which could indicate inadequate agreement. Bland-Altman analyses showed that, for most nutrients analyzed, the difference in reporting between the tools was not affected by the mean value of the intake. However, in addition to systematic bias, a significant trend was detected in iron reporting, where higher overall intakes correlated with higher Keenoa reporting. A slightly similar trend was also seen with folate and calcium, without systematic bias at the group level. Weighted Cohen κ scores confirmed fair (calcium, iron, sodium, and folate) to moderate (fiber and potassium) agreement at an individual level.

The difference between tools in estimating sodium intake may be explained by a combination of possible sodium overreporting with ASA24 and underreporting with Keenoa. First, neither ASA24 nor Keenoa asks participants about added salt. Although Keenoa does provide low- and no-sodium options for food items when applicable, ASA24 does not provide questions for salt adjustment and assumes that regular-sodium foods are consumed and that salt is added during food preparation [39]. Previous dietary intake surveys used to query on food items’ salt content and the addition of salt at the table. However, in a 2012 report by the United States Department of Agriculture, taking out those salt adjustment questions was shown to lower sodium intake estimates by about 4%. Thus, those questions were not included in the development of ASA24 [39]. ASA24 was found to overreport sodium intake in 1 study [19] but not in others [20,40]. Alternatively, the absence of questions related to added salt within Keenoa might have led to an underestimation of sodium intake. These differences may have exaggerated the difference in sodium estimation between the 2 tools.

Regarding fiber intake reporting, higher values with Keenoa seem to be associated with the inclusion of mixed meals in the food diaries of our participants. The Keenoa database includes meal builders which allow participants to select different items used to prepare their mixed meal. Each item is linked to their unique CNF code in a proportion that is representative of popular recipes for this specific meal in North America. For example, chili can be logged by selecting the specific protein items, vegetables, and toppings that constituted the meal and that were really consumed. In contrast, ASA24 uses the predefined mixed dishes included in the CNF database, which are often fast-food versions, containing less vegetables than homemade versions, or versions in which the meal content is standard and cannot be modified. Finally, the difference in iron reporting between the 2 tools was higher in frequent consumers of breakfast cereals, for which bar codes could be used with Keenoa to specify the brand and type of cereals consumed. For instance, entering “cereals” in ASA24 yields “cereal (cold, other kind)” and “cereal (unknown kind)” as first options, leading participants to be less specific about cereals consumed, whereas Keenoa allows these items to be easily entered while not providing the possibility for vague entries.

In our study, no gender difference was seen in the reporting agreement between tools, except for calcium intake, which was significantly different in men. The difference is relatively small (90 mg; 10% of mean estimated intake). This difference is not because of outlier data and remains unexplained. Upon inspection of data distribution, no significant outliers were detected, and differences are well distributed around the mean. The difference was not statistically significant when grouping all participants. In addition, when performing the analyses by age group, the same findings were obtained.

Current findings of overall agreement between tools differ from those of a previous relative validation study published in 2020 [27] in which Keenoa showed significantly lower reporting for energy and most nutrients when compared with a 3-day pen-and-paper food diary analyzed with the Food Processor software (version 11.1; ESHA Research), using the CNF food database. Contrasting findings between that study [27] and this study may be because of the use of a different reference method (3-day written food diary vs ASA24) but most likely related to upgrades made to the Keenoa app. The study by Ji et al [27] was conducted using a prototype (version 0.3.7) of the Keenoa app, which was restricted to a search for food items available within the CNF. This database is limited, most notably in its cultural food content, and thus limits participants from selecting some of their consumed food items. The version of Keenoa used in this study (version 1.0.3) had a larger database including cultural foods from other national databases and had integrated meal builders which potentially facilitated participants’ data entry. In addition, a multiple-pass function had also been implemented within Keenoa to maximize validity, prompting participants to review their diary at the end of the day and enter any omitted item. However, a conclusion as to which tool performs better cannot be verified as none of the 3-day food diary or ASA24 reference methods is a gold standard to estimate dietary intake.

Other mobile apps are available to estimate nutritional intake. Most of them are not adapted for research purposes, as they allow the public to freely add food items to the database [41] and thus databases are not validated [42]. Some are aimed to induce weight loss and are not adapted for dietary assessment as the energy and nutrient intakes are inevitably shown to the participant, possibly leading to changes in behavior and intake [41-46]. Some mobile apps used for research have not been adapted to the Canadian food market [47-49]. Image-assisted mobile dietary assessment methods also exist but some are not connected to a nutrient composition database and thus require researchers to review all entries [50], providing limited advantage compared with traditional methods. Others are not available to the public [51]. In addition, other relative validation studies on mobile tools are often performed on the same day of intake (food diary followed by a 24-hour recall the next day), which induces a major training bias. Results from those studies typically show limited systematic bias with wide LOAs [52], which is comparable with our study’s results even though our tracking periods were different between tools. Finally, some image-assisted methods involve wearable camera devices [53,54], which can also be time-consuming for researchers to collect and analyze data, in addition to potential ethical concerns and issues with participant acceptance because of their invasiveness.

Other web-based tools also exist to estimate nutritional intake in a recall format [20,55-58]. Most of them have been validated against recovery biomarkers and are currently used in research settings. Although most are adapted to European populations, 2 bilingual tools were available in Canada [20,57]. We selected ASA24 as a comparator to Keenoa because, in addition to being widely used in research, it has been extensively validated, notably against true intake [19], doubly labeled water, and urinary biomarkers [20]. However, ASA24 does have some limitations that might have limited adherence. First, it takes considerable time to complete the food recalls, between 41 and 58 minutes [40], given the automated multiple-pass method used to maximize validity. Second, because of the recall format, some participants were worried about forgetting their intake from the previous day and wrote down everything that was consumed in real time to properly enter it in ASA24 the next day, thus increasing the participant burden. The lower perceived usability with this tool led to the lower adherence that was detected with ASA24 in this study. This may lead to attrition bias where only the most motivated participants will report their intake or remain in longitudinal studies, thus limiting generalizability. It is noteworthy that participants who were assigned to start with ASA24 were more likely to drop out or be excluded from the study follow-up, consistent with the lower usability score from ASA24.

On the other hand, some participants preferred tracking once per day with ASA24 instead of continuously throughout the day with Keenoa. An advantage to ASA24 is the ability for prescription supplements to be entered and added to the nutritional analysis. The Keenoa app also has some limitations. For instance, it does not query participants on added salt, which might lead to sodium underreporting. The current Keenoa database does not include prescription vitamin and mineral supplements—those can only be added by hand by researchers or clinicians. However, the presence of a more extensive database, as well as the ability to copy previous meals, and the shorter time associated with tracking were preferred by participants, leading to a higher usability score. It is known that tracking periods over 4 days decreases the validity of filled diaries [5,59], likely secondary to a decrease in participant motivation. The perceived ease of use with the Keenoa app may increase food-tracking adherence over longer periods, thus leading to better estimations of one’s usual intake. This remains to be shown.

### Limitations

Our study has its limitations. Ideally, both measurement instruments should be used on the same days of food recording, but this approach would introduce training bias, as the use of one tool would influence the information provided with the other tool. Thus, we tested each instrument on 4 consecutive days, randomly, assuming a fairly constant intake of nutrients of interest. However, this approach increased intraindividual variability related to food intake, independently of the tracking tool. Furthermore, our sample was more educated and included a higher proportion of participants who self-identified as White than Canadian averages, which might limit the generalizability of our results. In addition, 11% (15/136) of our sample was trained in nutrition or dietetics and 19.9% (27/136) had type 1 diabetes, which may have improved the quality of food tracking. However, comparing a food recall to a food diary could have been leading to some differences in results between tools. We also aimed to recruit more participants, specifically men, and participants with diabetes. Despite numerous efforts to complete our group quotas, we experienced a plateau in recruitment. As the group having diabetes was inadequately powered to detect significant correlations and the results from this group did not differ from those of the healthy group, we pooled both groups together to ensure an adequate sample size. Finally, although extensively validated, ASA24 is not a gold standard measure. It is thus not possible to identify which tool provided results that were most representative of true intake. This supports the continuation of the validation process into a validation with recovery biomarkers or true intake measures.

### Conclusions

The Keenoa app showed moderate to strong relative validity against ASA24 for energy, macronutrient, and most micronutrient intakes analyzed in healthy adults and those living with diabetes. Keenoa is a new, alternative tool that may facilitate the work of dietitians and researchers in nutrition. The perceived ease of use may improve food-tracking adherence over longer periods and minimize attrition bias.

## Appendix

Multimedia Appendix 1 Baseline questionnaire and deattenuated correlation coefficients.

## Abbreviations

ASA24: automated self-administered 24-hour recall
BETTER: Behaviors, Therapies, Technologies, and Hypoglycemic Risk in Type 1 Diabetes
CNF: Canadian nutrient file
LOA: limit of agreement
SUS: System Usability Scale
