# Supplementary material for: Relative Validation of an Artificial Intelligence–Enhanced, Image-Assisted Mobile App for Dietary Assessment in Adults: Randomized Crossover Study
Source: J Med Internet Res. 2022 Nov 21;24(11):e40449. doi: 10.2196/40449 (PMC9723975; doi:10.2196/40449)
Supplement: Multimedia Appendix 1 [file jmir_v24i11e40449_app1.doc]

Multimedia appendix 1. Participant questionnaire

1. Participant ID :

___________

1. What is your date of birth (year/month/day)?

___________

1. Which sex was attributed to you at birth?
   1. Male
   2. Female
   3. I prefer not to answer
2. What gender do you identify with?
   1. Man
   2. Woman
   3. Other
   4. I prefer not to answer
   5. I don’t know
3. Do you have a background or are your currently training in dietetics or human nutrition?
   1. Yes
   2. No
4. What is your employment status? Check all that apply.
   1. Full-time
   2. Part-time
   3. Unemployed
   4. Student
   5. Volunteer
   6. Caregiver
   7. Retiree
   8. Occasional or seasonal
   9. Self-employed
   10. I don’t know
   11. I prefer not to answer
5. Indicate the highest level of education you acquired.
   1. Some high school completed
   2. High school diploma
   3. Vocational school
   4. Completed CEGEP
   5. Bachelor’s degree
   6. Graduate degree (ie. MSc, MA, PhD)
   7. Professional degree (ie. MD)
   8. I prefer not to answer
   9. I don’t know
6. Indicate the primary ethnicity which you belong to.
   1. White/Caucasian
   2. Aboriginal (First Nations)
   3. East Asian (ie. Chinese, Filipino, Korean, Japanese, Vietnamese)
   4. South Asian, West Asian or Middle Eastern (ie. Arab, East Indian, Pakistani, Iranian)
   5. Black
   6. Hispanic
   7. Mixed
   8. I don’t know
   9. I prefer not to answer
7. Are you currently living with one or more chronic disease(s)?
   1. Yes
   2. No
   3. Unsure
   4. I don’t know
   5. I prefer not to answer
8. Have you ever been diagnosed with the following chronic disease(s)? Please select all that apply.
   1. Hypertension
   2. Osteoarthritis
   3. Asthma
   4. Cancer
   5. Mood or anxiety disorder
   6. Chronic Obstructive Pulmonary Disorder (COPD)
   7. Heart disease
   8. Stroke
   9. Diabetes
   10. Dementia or Alzheimer’s disease
   11. Other (specify)
   12. I prefer not to answer
   13. I don’t know

**Conditional: If answered 10 k)**

1. If you selected other, please specify.

________

1. This next question concerns your height. Do you want to give your height in feet or meters?
   1. Feet
   2. Meters
   3. I don’t know
   4. I prefer not to answer

**Conditional: If answered 11 b)**

1. What is your current height (meters)?

__________

**Conditional: If answered 11 a)**

1. What is your current height (feet)?

__________

**Conditional: If answered 11 a)**

1. Add the inches here.

__________

1. This next question concerns your weight. Do you want to give your weight in pounds or kilograms?
   1. Pounds
   2. Kilograms
   3. I don’t know
   4. I prefer not to answer
2. What is your current weight?

________

1. Is your current weight different from your usual body weight?
   1. Yes
   2. No
   3. I don’t know
   4. I prefer not to answer

**Conditional: If answered 17 a)**

1. What is your usual body weight?

________

1. When is the last time you were at your usual body weight?

________

1. Have you ever experienced weight loss of more than 5kg or 10 pounds?
   1. Yes
   2. No
   3. I don’t know
   4. I prefer not to answer

**Conditional: If answered 21 a)**

1. How many times have you experienced weight loss of more than 5 kg or 10 pounds?
   1. Once
   2. 2 times
   3. 3 times
   4. More than 3 times
   5. I don’t know
   6. I prefer not to answer
2. Are you currently satisfied with your body weight?
   1. Yes
   2. No
   3. I don’t know
   4. I prefer not to answer

**Conditional: If answered 23 b)**

1. What is the primary reason you selected no to the previous question?

This question asks why you are dissatisfied with your body weight.

- 1. I want to lose weight
  2. I want to gain weight
  3. I receive negative comments about my body’s appearance
  4. I don’t know
  5. I prefer not to answer

1. In the past week have you had any surgeries (including dental), or other medical procedures?
   1. Yes
   2. No
   3. I don’t know
   4. I prefer not to answer
2. Please select all of the medications you are currently taking.
   1. No medication
   2. Thyroid medication
   3. Blood pressure medication
   4. Cholesterol medication
   5. Analgesics
   6. Anti-coagulants
   7. Proton Pump Inhibitors
   8. Anti-depressants
   9. Bronchodilators
   10. Diuretics
   11. Insulin Therapy
   12. Oral antihyperglycemic agents (ie. metformin, Thiazolidinediones, GLP-1 receptor agonists)
   13. Attention deficit disorder or attention deficit hyperactive disorder medication
   14. Other (specify)
   15. I don’t know
   16. I prefer not to answer

**Conditional: If answered 26 k)**

1. Which insulin medication(s) are you on?
   1. NovoRapid
   2. Apidra
   3. Humalog (U-100, U-200)
   4. Fiasp
   5. Humulin-R Novolin ge Toronto
   6. Entuzity (U-500)
   7. Humulin N, Novolin ge NPH
   8. Levemir
   9. Lantus
   10. Toujeo
   11. Basaglar
   12. Tresiba
   13. Humulin 30/70
   14. Novolin ge 30/70, 40/60 or 50/50
   15. Novomix 30
   16. Humalog Mix25 or Mix50
   17. Other (specify)

**Conditional: If answered 26 n)**

1. If you've selected other, please specify:

________

**Conditional: If answered 26 l)**

1. Which oral anti-hyperglycaemic agent(s) are you currently taking?
   1. Acarbose (Glucobay)
   2. Repaglinide (Prandin)
   3. Glyburide (Diabeta)
   4. Sitagliptin (Januvia)
   5. Exenatide (Byetta)
   6. Liraglutide
   7. Pioglitazone (Actos)
   8. Rosiglitazone (Avandia)
   9. Canagliflozin
   10. Dapagliflozin
   11. Empagliflozin
   12. Other (specify)

**Conditional: If answered 27 q)**

1. Please specify which insulin therapy you currently take.

________

**Conditional: If answered 29 l)**

1. Please specify which anti oral-hyperglycaemia medications you currently take.

________

1. Please select all of the nutritional, vitamin and mineral supplements you are currently taking.
   1. No supplements
   2. Complete Meal supplements (shakes, bars, pudding)
   3. Protein supplements (powder or shakes)
   4. Medicinal herbs
   5. Special oils (ie. Fish)
   6. Multivitamin and mineral
   7. Vitamin D
   8. Calcium
   9. Omega-3
   10. Folic acid
   11. Iron
   12. Zinc
   13. Magnesium
   14. Potassium
   15. Vitamin C
   16. Vitamin E
   17. Other (specify)
   18. I don’t know
   19. I prefer not to answer

**Conditional: If answered 32 q)**

1. If you’ve selected other, please specify.

________

**Conditional: If answered 32 any response besides a), r), and s)**

1. Please specify the dose of all supplements selected.

________

1. Would you say your current diet is representative of your usual diet?
   1. Yes
   2. No
   3. Unsure
   4. I don’t know
   5. I prefer not to answer
2. Please select all special diets which you are following. If not applicable, please select N/A.
   1. N/A
   2. Ketogenic
   3. Low carb
   4. Mediterranean
   5. Intermittent fasting
   6. Paleo
   7. Whole 30
   8. Veganism
   9. Vegetarianism
   10. Raw food
   11. Gluten-free
   12. Other (specify)
   13. I prefer not to answer
   14. I don’t know

**Conditional: If answered 36 l)**

1. If you’ve selected other, please specify.

________

1. Who is primarily responsible for the cooking at your home?
   1. Myself
   2. Another family member
   3. My roommate
   4. My caregiver
   5. My housekeeper
   6. I don’t know
   7. I prefer not to answer
2. Who is primarily responsible for the grocery shopping at your home?
   1. Myself
   2. Another family member
   3. My roommate
   4. My caregiver
   5. My housekeeper
   6. I don’t know
   7. I prefer not to answer
3. How often do you eat our or order food from a restaurant? (Including fast food).
   1. More than 7 times per week
   2. 5-6 times per week
   3. 3-4 times per week
   4. 1-2 times per week
   5. Less than 1 time per week
   6. I don’t know
   7. I prefer not to answer
4. Is anyone else in your household participating in this study?
   1. Yes
   2. No
5. Please select any diet tracking app which you’ve utilized previously.
   1. N/A
   2. MyFitnessPal
   3. Fitbit
   4. Lose It!
   5. Lifesum
   6. Calorie Counter
   7. My Diet Coach
   8. Other (specify)

**Conditional: If answered 42 h)**

1. If you’ve selected other, please specify.

________

1. Please select your typical daily activity
   1. Chair or bed bound
   2. Seated work with little movement, and little to no leisure activity
   3. Seated work with requirement to move, little strenuous activity
   4. Standing work
   5. Strenuous work or highly active work
2. In addition to your daily activity indicated above, do you also engage in 30-60 minutes of strenuous leisure activity at least 4-5 times per week?
   1. Yes
   2. No
3. Thinking about your level of physical activity, are you:

Sedentary: office worker with little or no exercise

Low active: Sedentary occupation but walks about 3 km/day

Active: Sedentary occupation with planned moderate to vigorous exercise for 1 hour/ day ie. running, cycling, or swimming.

Very active: Non-sedentary occupation and moderate to vigorous exercise for >2 hours per day

1. Sedentary
2. Low active
3. Active
4. Very active
5. During the last 7 days, did you do vigorous physical activities like heavy lifting, digging, aerobics, or fast bicycling?

Vigorous physical activities refer to activities that take hard physical effort and make you breathe much harder than normal. Think only about those physical activities that you did for at least 10 minutes at a time.

- 1. Yes
  2. No
  3. I don’t know/Not sure

**Conditional: If answered 47 a)**

1. During the last 7 days, on how many days did you do vigorous physical activities like heavy lifting, digging, aerobics, or fast bicycling?
   1. 1
   2. 2
   3. 3
   4. 4
   5. 5
   6. 6
   7. 7
   8. 0

**Conditional: If answered any of 48 a) – g)**

1. Do you know how much time you usually spend doing vigorous physical activities on one of those days?
   1. Yes
   2. Don’t know/Not sure

**Conditional: If answered 49 a)**

1. How much time (in minutes) did you usually spend doing vigorous physical activities on one of those days?

________

1. During the last 7 days, did you do moderate physical activities like carrying light loads, bicycling at a regular pace, or doubles tennis? Do not include walking.

Moderate activities refer to activities that take moderate physical effort and make you breathe somewhat harder than normal. Think only about those physical activities that you did for at least 10 minutes at a time

- 1. Yes
  2. No
  3. Don’t know/Not sure

**Conditional: If answered 51 a)**

1. During the last 7 days, on how many days did you do moderate physical activities like carrying light loads, bicycling at a regular pace, or doubles tennis? Do not include walking.
   1. 1
   2. 2
   3. 3
   4. 4
   5. 5
   6. 6
   7. 7
   8. 0

**Conditional: If answered any of 51 a) – g)**

1. Do you know how much time you usually spend doing moderate physical activities on one of those days?
   1. Yes
   2. Don’t know/Not sure

**Conditional: If answered 53 a)**

1. How much time (in minutes) did you usually spend doing moderate physical activities on one of those days?

________

1. During the last 7 days, did you walk for at least 10 minutes at a time?

This includes at work and at home, walking to travel from place to place, and any other walking that you have done solely for recreation, sport, exercise, or leisure.

- 1. Yes
  2. No
  3. Don’t know/Not sure

**Conditional: If answered 55 a)**

1. During the last 7 days, on how many days did you walk for at least 10 minutes at a time?
   1. 1
   2. 2
   3. 3
   4. 4
   5. 5
   6. 6
   7. 7
   8. 0

**Conditional: If answered any of 56 a) – g)**

1. Do you know how much time you usually spend doing walking on one of those days?
   1. Yes
   2. Don’t know/Not sure

**Conditional: If answered 57 a)**

1. How much time (in minutes) did you usually spend walking on one of those days?

________

1. Do you know how much time you spend sitting on a weekday?

This includes time spent at work, at home, while doing course work and during leisure time. This may include time spent sitting at a desk, visiting friends, reading, or sitting or lying down to watch television

- 1. Yes
  2. No
  3. Don’t know/Not sure

**Conditional: If answered 59 a)**

1. How much time (in minutes) did you usually spend sitting on a weekday?

________

1. I would like to receive an evaluation of my diet
   1. Yes
   2. No
2. I authorize a member of the research study to contact me in the future to ask if I am interested in participating in follow-up studies related to this research.
   1. Yes
   2. No
3. If you’d like to be entered to win $100, please provide the answer to this mathematical equation to be considered in the raffle.

(4+10) ÷ 2

________

Supplemental Table 1. Deattenuated correlation coefficients for ASA24 and Keenoa reporting.

| **Nutrients** | **Females**  **N=87** | | **Males**  **N=49** | |
| --- | --- | --- | --- | --- |
|  | ρ | *P* value | ρ | *P* value |
| **Energy**(kcal) | 0.717 | <0.001** | 0.851 | <.001** |
| **Carbohydrates**(g) | 0.842 | <0.001** | 0.888 | <.001** |
| **Protein**(g) | 0.420 | 0.01** | 1.000^a^ | <.001** |
| **Fat**(g) | 0.715 | <0.001** | 0.689 | <.001** |
| **Fiber** (g) | 0.936 | <0.001** | 0.988 | <.001** |
| **Calcium** (mg) | 0.735 | <0.001** | 0.769 | <.001** |
| **Iron** (mg) | 0.767 | <0.001** | 0.958 | <.001** |
| **Sodium** (mg) | 0.307 | 0.093 | 1.000^a^ | <.001** |
| **Potassium** (mg) | 1.000^a^ | <0.001** | 0.730 | <.001** |
| **Folate** (mcg) | 0.691 | <0.001** | 0.612 | .001** |

Deattenuated spearman correlation coefficients

^a^CC>1.0; could either indicate that the correlation was strong before adjustment, or that daily intraindividual variability was high with one or both tools, leading to low reliability for that nutrient.
